# Exploratory Study of the Relationship Between Happiness and the Rise of Media Consumption During COVID-19 Confinement

**DOI:** 10.3389/fpsyg.2021.566517

**Published:** 2021-04-29

**Authors:** José Antonio Muñiz-Velázquez, Diego Gómez-Baya, Javier Lozano Delmar

**Affiliations:** ^1^Department of Communication and Education, Universidad Loyola Andalucía, Seville, Spain; ^2^Department of Social, Developmental and Educational Psychology, Universidad de Huelva, Huelva, Spain

**Keywords:** media consumption, well-being, happiness, confinement, COVID-19

## Abstract

The confinement of the population into their homes as a result of COVID-19 has entailed a notable increase in the consumption of diverse media. This exploratory study aimed to examine how the increase in media consumption was related to subjective happiness and psychological well-being. For this purpose, a questionnaire was administered to a sample of Spanish adults (*n* = 249; 53.8% women; aged between 18 and 75, *M*_age_ = 42.06, *SD* = 12.37) to assess their consumption of different media before and during confinement. Moreover, participants were evaluated for hedonic, eudaimonic, social, and experienced happiness by using the *Pemberton Happiness Index* (PHI). The results underlined the great increase in the consumption of TV for entertainment and social networking sites (SNS) during confinement. Furthermore, it was found that higher consumption was negatively correlated with the level of happiness, so that, people who reported greater well-being, both subjective and psychological, spent less time watching TV and using SNS. In contrast, no association was found between the level of happiness and the consumption of news (regardless of the media) and radio. Therefore, it seems that far from cultivating greater happiness, those who engaged in heavy consumption of TV entertainment and SNS during confinement were less happy than those who did so more moderately and spent more time using other media or performing other activities.

## Introduction

The main tragedy of COVID-19 has been the death and illness of millions of people around the world. But, the pandemic has also led to an unprecedented situation for most of the world population: home confinement for many weeks at a time. Thus, individuals and families across were suddenly forced to rethink a daily routine to be carried out entirely inside their homes. It was necessary to reconfigure the daily habits that characterized the previous normal life. During quarantine period, some daily behaviors disappeared, while new ones would emerge, and in most cases their duration changed.

Media consumption was one of the habits with a greater increase during confinement. Various studies confirm this result (Casero-Ripollés, 2020), as well as reports from the media industry, such as the Spanish Association for Media Research (AIMC), who measured media consumption during confinement through various waves of weekly monitoring. For instance, the second report during the week of 13–19 April (the 5th week of confinement in Spain) shows an increase in the consumption of digital magazines and radio. Furthermore, in the third assessment (week from 20 to 26 April), it was observed that “radio exceeds 20.5 million listeners per day with an increase in all time slots, especially from 6 to 10 in the morning” (AIMC, 2020a). In addition, “62% of Internet users have increased their viewing time for both free-to-air and pay channels to combat boredom [and] subscriptions to OTTs continue to grow with a 7.6% increase in recent weeks” (AIMC, 2020b).

Along the same lines, the study of Comscore (2020) pointed out an increase in media consumption in several European countries, i.e., France, the United Kingdom, Germany, Italy and Spain. In these countries, the consumption of contents such as general information and local news apps rose, specifically during the months of March and April. The same study also showed a similar peak in the use of instant messaging applications and social networks during those same months of the year 2020.

Barlovento Comunicación (2020) released another report highlighting the historical record of Internet and TV consumption in Spain, with 2 h and 56 min per person/day in the case of web access (an increase of 37 min compared to the same month in 2019) and an average TV consumption of 5 h and 10 min. This study coincides with Kantar consulting findings (Nafría, 2020), which suggest a sustained growth in TV consumption exceeding 40%. Kantar’s results have highlighted the time devoted to news, both in terms of reach (33% more) and intensity, reflected by the time allotted to this type of content more than doubling. In this sense, Masip et al. (2020) and Rodero (2020) argued that in crisis situations, audiences tend to focus on traditional media, especially TV, followed by radio. However, this crisis appears to involve a great increase also in social network use.

According to data from the latest report prepared by Hootsuite and “We are social” (Fernández, 2020), in Spain, 47% of Internet users said they were spending more time on social networks and 23% of them said that they spent “much more” time on the networks, compared to their pre-quarantine habits. As the report indicates, social networking site (SNS) had a significant rise in their number of active users during the first quarter of 2020. The greatest growth occurred in Twitter, with an increase in user about 14% (Fernández, 2020). In Spain, the platform preferred during confinement was Instagram, which has reported an increase of 6% in active users (more than 1 million people). Thus, Spain was the third European country in terms of its activity increase on this network during the COVID-19 crisis (Fernández, 2020).

The resurgence of the hegemony of TV during the pandemic (Casero-Ripollés, 2020) is not a great surprise, given that, it remained one of the most widespread leisure activities even before confinement (Frey et al., 2007; Frey, 2018). However, the increase in TV consumption during confinement was paradoxical, considering that the vast majority of scientific evidence (Robinson and Martin, 2008) suggests an inverse relationship between excessive TV consumption and individual happiness.

Psychological studies on the effects of TV on the well-being and health of the audience have a long tradition, with the work by Argyle and Lu (1992) as pioneer. In this line, special attention has been paid to children (Hamer et al., 2009; McDade-Montez et al., 2015), reaching certain consensus on the harmful effect that excessive hours of TV may have on both child physical and mental health. Studies on the adult population show similar results, as well. Thus, Lu and Argyle (1993) found that TV consumption in general was associated with lower happiness, as measured by the Oxford Happiness Inventory (OHI), although specific consumption of Soap Operas correlated with higher happiness. The authors warned, however, about the possible mediation of personality differences in such results. In other study, Hills and Argyle (1998) examined the same relationship between viewing TV Soap Opera and happiness, and found no significant relationship. Furthermore, Frey et al. (2007) found that intensive TV users in general reported lower life satisfaction, more material aspirations and a higher level of anxiety. Cuñado and Pérez de Gracia (2012) also showed an association between TV consumption and negative affect.

It should be noted that TV consumption can be very heterogeneous, given the variety of broadcast content, as Gui and Stanca (2009) underlined. These authors pointed out the need to highlight qualitative aspects in the studies exploring the relationship between TV consumption and well-being. Thus, Kim et al. (2017) focused on viewing live sports events. As these authors suggested, well-being improves when hedonic, eudaimonic, and social needs are satisfied, and watching sports on TV seemed to be favorable for all of them. Sports broadcasting may involve different characteristics compared to other TV contents. However, live sports on TV were not available during confinement.

There was a controversy concerning causality in the relationship between those two variables, as Bayraktaroglu et al. (2019) highlighted. Many studies have found a negative interrelation between TV viewing time and different indicators of well-being. However, for these authors, causality is not clear in that previous evidence. Moreover, they advocate an inverse causal relationship: lower happiness, in hedonic terms, may be the cause for more TV watching, rather than increased TV watching causing unhappiness. As the same authors said, people try to distract themselves with TV expecting to feel better, so unhappiness would be what causes the increase in TV consumption. In any case, such consumption does not seem to significantly improve hedonia neither for a desirable period, but may have a detrimental effect.

In any case, until now the relationship between both variables, hours of TV and happiness, have been studied in normal conditions, and not in such an exceptional and unprecedented situation, nor under the widespread uncertainty in social, economic, and health terms of a worldwide pandemic. Therefore, it seemed more than pertinent to observe if the relationship between the two variables might vary in such a unique scenario. Moreover, it is also interesting to examine the separate effect by general entertainment on TV and fiction-related (series and movies) consumption from news’ consumption. Concerning the consumption of series and movies on TV, it is possible to find a source of psychological well-being that goes far beyond mere audiovisual entertainment (Oliver and Bartsch, 2010). Fiction series and movies, as cultural products, can also provide an intellectual and cognitive stimulation, i.e., it can become an eudaimonic entertainment source, and not just a hedonic one (Vorderer and Reinecke, 2015; Lozano Delmar et al., 2018; Oliver and Raney, 2019).

Furthermore, regarding the increase in news consumption, it is expected an inverse relationship with the level of happiness, regardless of the media. In a situation like the current one, where most of the news are negative and directly related to the pandemic and its consequences, it seems plausible to think that a greater and intensive monitoring of news could have adverse effects on the happiness of the audience. This is shown by studies not only previous to the pandemic (Johnston and Davey, 1997; Havrylets et al., 2013) but also studies conducted during confinement (Masip et al., 2020).

In contrast, the starting point for radio was different from that of TV. Although its relationship with happiness may be detrimental, the mass media par excellence has always demonstrated a leading role in times of crisis, as Rodero (2020) and others remind us. It is worth noting that radio, due to its versatility and technological simplicity compared to other mass media, has been a preferential mean of information in turbulent times. In addition to informing, radio has a traditional power to comfort and to provide companionship. Thus, it is considered the most intimate media, partly due to the “particularly intense sense of presence that radio possesses” (Karathanasopoulou, 2014, p. 97). In relation to radio consumption during confinement, it may be expected a positive relationship between increased consumption time and happiness, or at least, no decrease in happiness, as pointed out Cuñado and Pérez de Gracia (2012). It is important to note that the radio, by its acoustic nature, enables doing something else while listening. Consequently, the effects of its consumption on well-being can be mediated by other simultaneous activities with listening.

With regard to the use of SNS and their relationship with happiness, some studies have shown a variety of results. Among those studies which indicated a positive effect of social networks on the users’ well-being, Chan (2018) observed a positive relationship between subjective well-being (SWB) with the number of friends or contacts on Facebook, especially among young people. Hu et al. (2017) also noticed some benefits of using Facebook, in terms of psychological well-being, although they were conditioned by the online-offline social contexts and personality characteristics (with stronger relationship among introvert people). Huang (2016) detected some beneficial effects of individual self-disclosure on SNS through social support and online social well-being. In the same line, Gilmour et al. (2019) conducted a review of studies on Facebook and its association with social support and health. They found that overall Facebook-based social support predicted better outcomes in both mental and physical health, although they also found considerable exceptions. They acknowledged that the study was not about general Facebook use, but about whether users seek and find social support through Facebook. Similarly, Clark et al. (2018) argued that, if used to make meaningful social connections, SNS can be beneficial to well-being. However, these authors also warned about the danger of becoming a trap of isolation and social comparison, which that are not conducive to happiness (Smith et al., 1989; Yamada and Takahashi, 2011).

Thereby, Liu and Yu (2013) argued that although the use of Facebook certainly implies a perception of greater social support, this is weakly linked to well-being, because this relationship is mediated by general social support, received outside SNS. Lima et al. (2017) highlighted the positive effects of online friendships compared to face-to-face relationships. Furthermore, Arampatzi et al. (2018) established that online social contacts will never replace the role and prominence of real-life social contacts in the human pursuit of happiness. But apart from the specific usage profile in qualitative terms, the quantitative increase in SNS usage time seems clearly detrimental on well-being. Besides the fact that personal and cultural variables may mediate online behavior and its psychological consequences (Castellacci and Tveito, 2018), most recent studies are concluding, i.e., more time spent on SNS is associated with a lower level of happiness (Arampatzi et al., 2018; Faelens et al., 2021). Only a study, among teenage students in Turkey (Dogan et al., 2018), presented a positive relationship between time spent using SNS (Facebook and Twitter) and increased happiness. In contrast, Twenge (2019) concluded that while moderate use of SNS could be beneficial, an excessive increase is clearly negative to well-being. In the same vein, Frost and Rickwood (2017) found in their meta-analysis that intense use of Facebook could be associated with mental health problems such as anxiety, depression, addictions, or eating disorders. Andreassen et al. (2016) or Tang et al. (2016) or Hussain and Griffiths (2018) found a strong association between problematic use of SNS and symptoms of psychiatric disorders, especially among adolescents. Consequently, it seems unlikely that this increase in SNS time during confinement would imply a higher level of happiness or well-being.

So, following the studies mentioned above, the aim of this work was to examine the relationship between media consumption and psychological well-being in a new and ever seen situation due to the confinement during Spring 2020 in Spain. Given that media consumption experienced a huge growing during that period of confinement (Casero-Ripollés, 2020), which in Spain began in mid-March of the year 2020, it can be assumed that people increased their media consumption in order to find a source of some kind of well-being, if not eudaimonic, at least hedonic, during their daily confinement at home.

## The Present Research

The aim of the present study was to explore whether the supposed increase in media consumption, as several sources have already indicated, during the weeks of confinement at home because of the COVID-19 pandemic was positively or negatively related to the level of happiness and well-being. First, it is aimed to study if there was in fact a significant increase in the use of the different media. Second, the relationship between media consumption and happiness was explored, as well as the relationship between the change in media consumption during confinement and the levels of happiness. On the basis of previous literature, the hypotheses were:

H_1_: Higher daily consumption of audiovisual fiction (TV series and movies) and higher increase compared to pre-confinement are expected to be associated with greater happiness.

H_2_: Higher daily TV consumption (general entertainment) and higher increase in consumption compared to pre-confinement, are expected to be associated with lower happiness.

H_3_: Higher daily news consumption and higher increase in consumption compared to pre-confinement, are expected to be related to lower happiness.

H_4_: Greater daily radio consumption and greater increase in the consumption during confinement, are expected to be related to be associated with greater happiness.

H_5_: Greater daily consumption of social networks (SNS) and greater increase during confinement are expected to be associated with lower happiness.

### Sample and Procedure

A questionnaire was administered using Qualtrics,^1^ and distributed through different digital channels and networks (WhatsApp, Twitter, Facebook, and mailing). The sample was composed of 249 adults (53.8% women) aged between 18 and 75 (*M*_age_ = 42.06, *SD* = 12.37), by carrying out a non-probabilistic snowball sampling procedure (the participants helped to share the link of the online survey through their own SNS). Most participants were Spanish (99.1%), did not lose their job (91.9%), did not contract the virus (95.1%), and did not lose a loved one (95.9%). Furthermore, 72.2% knew someone infected by the virus. In terms of social comparison of the situation during this confinement (“In general and compared to other citizens, how do you think that your situation is during pandemic?”), 4.5% of the respondents reported their situation as being worse than average, 26.1% indicated that their situation was similar to average, while 69.4% indicated being better than average. Responses, collected in April and May of the year 2020, were anonymous and confidential, since no personal data were requested to identify the participant.

The questionnaire comprised different sections. First, some socio-demographic questions were included, as well as employment and contextual variables. These questions covered both generic items and those related to the particular pandemic situation. Second, the subjects were asked about their average media consumption time, estimated in minutes per day. The question (and the answer) was double, referring to the time spent before confinement, and the time spent during confinement (e.g., *How many minutes on average per day did you dedicate before confinement and now during the confinement dedicate yourself to listen to the radio?*) Time scale ranged from a daily average of 0–150 min or more, with the more common intervals that are used in daily life (up to 10 min/around 15 min/around 20 min/around 30 min/around 40 min/around 45 min/around 50 min/around 1 h/around 1 h and a quarter/around 1 h and a half/around 1 h and three quarters/around 2 h/around 2 h and a half or even more).

In relation to TV, participants were separately asked about their consumption of generic entertainment, and fiction content (series and movies), while the consumption of news was asked regardless of the media. Regarding the use of radio and SNS, they were asked about time spent on both without distinguishing the nature of the content.

There are numerous instruments for measuring happiness and well-being (Cooke et al., 2016; Frey, 2018). Each one emphasizes certain aspects over others, according to the theoretical approach (see Veenhoven, 2017). Thus, it is interesting to take into account the two great dimensions of human happiness: hedonia and eudaimonia. They continue to have elusive and multifaceted definitions (Huta, 2013). The first one could be summarized as the subjective well-being that implies a generalized life satisfaction, where positive emotions are more prevalent than negative ones. The latter can be defined as that psychological well-being that stems from an optimal and purposeful life, where a sense of vital fulfillment prevails, while maintaining the ethical sphere of the human being. It is worth remembering that both play a complementary role in the overall human happiness (Huta, 2015), so that they cannot be fully understood without each other.

An instrument was used to equally cover both dimensions and not being excessively long and complex, given the conditions of online administration. Pemberton Happiness Index (PHI; Hervás and Vázquez, 2013), was used, because tool it has already demonstrated its cross-cultural validity (Ribeiro Paiva et al., 2016; Wade et al., 2018). This scale provides a measure of complete well-being, assessing both hedonia and eudaimonia, and also a social dimension and experienced happiness. For example, with items such as “Yesterday, I felt satisfied by something I did,” or “Yesterday, I allowed myself a whim,” with concrete actions from the previous day, of special interest for the study during the confinement situation, where monotony might be problematic. Overall, PHI has 11 items following a 11-point Likert scale, and 10 dichotomous items, similar to those already outlined. Table 1 collects the descriptive statistics of happiness dimensions and overall PHI index. Results showed notable scores in happiness dimensions, with the highest mean found in eudaimonic well-being and the lowest one, on social well-being. The overall PHI index reached a noteworthy mean score, with 7.68 (*SD* = 1.26) over a maximum of 10. Regarding reliability, overall scale presented excellent internal consistency (*α* = 0.89). As well, excellent reliability was also observed in the dimensions: general well-being (*α* = 0.76), eudaimonic well-being (*α* = 0.87), and hedonic well-being (*α* = 0.90).

**Table 1:** Descriptive statistics of happiness dimensions.

	Minimum	Maximum	*M*	*SD*
General well-being	2.50	10	7.64	1.60
Eudaimonic well-being	1.33	10	8.06	1.32
Hedonic well-being	0	10	7.36	1.85
Social well-being	0	10	6.42	2.16
Experienced well-being	2.00	10	7.37	1.78
PHI	3.42	10	7.68	1.26

### Data Analysis Design

First, descriptive statistics (i.e., mean and SD) of habits (i.e., watching TV: series and movies; watching TV: general; following news; listening to the radio; using social network sites; and participating in fan communities), before and during confinement were studied. Second, repeated measures variance analyses were conducted to examine change in habits after and during confinement, calculating partial eta squared as size effect indicator.

Third, Pearson bivariate zero-order correlations were calculated to analyze the associations between the frequency of habits during confinement and the scores in happiness dimensions and PHI index. Confidence intervals for correlations were also calculated. Fourth, correlation analyses were also carried out to assess the interrelations between the change in habits during confinement and happiness. The variables of change were determined by calculating the difference between the frequency of each habit during confinement and before confinement. These statistical analyses were all conducted using SPSS 21.0.

## Results

### Descriptive Statistics of Media Consumption

Table 2 presents means in minutes per day spent on the aforementioned habits before and during confinement. Concerning habits before confinement, participants reported having spent more minutes a day watching series and movies on TV, watching other contents on TV, and using social networks. During confinement, these same habits were also the most frequent, followed by keeping up with the news. The lowest mean scores, before and during confinement, were detected regarding participation in fan communities.

**Table 2:** Descriptive statistics of habits before and during confinement, and analysis of the change.

	Before confinement		During confinement		Change analysis	
	*M*	*SD*	*M*	*SD*	*F*	*η*^2^_p_
Watching TV: series and movies	53.27	36.57	83.56	49.78	131.53^***^	0.37
Watching TV: general	40.86	41.22	62.61	53.22	78.56^***^	0.26
Listening to the radio	34.57	39.85	33.27	47.33	0.29	0.01
Following news	33.76	28.12	53.06	37.19	84.91^***^	0.28
Using SNS (Facebook, Instagram…)	41.27	36.76	63.19	49.53	117.78^***^	0.35

*** *p* < 0.001.

### Analysis of the Change in Habits During Confinement

Table 2 also reflects the analysis of the change in the minutes spent in each habit before and during confinement. Significant increases in the frequency were observed in watching TV (series, movies, and general entertainment), following news, using social networks. The time spent in watching TV series and movies increased the most, around half hour during confinement. Furthermore, an increase of around 20 min was observed in social network use, as well as watching general contents and following news. No remarkable changes were found in listening to the radio nor in participating in a fan community.

### Associations Between Habits During Confinement and Happiness

Table 3 shows bivariate correlations between habits during confinement and happiness dimensions and overall PHI index. PHI scores were negatively associated with watching TV, both series/movies and general contents, with using social networks and creating/sharing contents online. Watching series and movies was negatively related to all types of well-being, i.e., general, eudaimonic, hedonic, social, and experienced. Furthermore, watching TV in general presented negative associations with both hedonic and social well-being. Using social networks was negatively associated with general, hedonic, and experienced well-being. Finally, no significant correlations were detected between happiness’ indicators and the habits of listening to the radio and following news.

**Table 3:** Pearson bivariate correlations between habits during confinement and happiness dimensions.

	General well-being	Eudaimonic well-being	Hedonic well-being	Social well-being	Experienced well-being	PHI
Watching TV: series and movies	−0.14^*^ (−0.28, −0.01)	−0.14^*^ (−0.27, −0.02)	−0.30^***^ (−0.42, −0.17)	−0.17^*^ (−0.29, −0.03)	−0.18^**^ (−0.31, −0.04)	−0.22^**^ (−0.35, −0.09)
Watching TV: general	−0.12 (−0.26, 0.03)	−0.12 (−0.24, 0.02)	−0.18^**^ (−0.31, −0.04)	−0.14^*^ (−0.26. −0.01)	−0.05 (−0.17, 0.09)	−0.16^*^ (−0.28, −0.01)
Listening to the radio	0.11 (−0.01, 0.21)	0.07 (−0.03, 0.18)	0.11 (−0.01, 0.20)	0.05 (−0.08, 0.17)	0.05 (−0.08, 0.18)	0.10 (−0.01, 0.21)
Following news	−0.06 (−0.20, 0.09)	−0.05 (−0.18, 0.09)	−0.13 (−0.27, 0.03)	0.03 (−0.13, 0.18)	−0.11 (−0.26, 0.02)	−0.08 (−0.22, 0.07)
Using SNS	−0.21^**^ (−0.34, −0.06)	−0.12 (−0.26, 0.01)	−0.21^**^ (−0.33, −0.08)	−0.06 (−0.20, 0.08)	−0.28^***^ (−0.41, −0.15)	−0.20^**^ (−0.33, −0.07)

* *p* < 0.05;

** *p* < 0.01;

*** *p* < 0.001.

### Associations Between Change in Habits During Confinement and Happiness

Table 4 describes the bivariate correlations between the changes in consumption habits comparing before and during confinement and happiness indicators. Results showed that greater increase in watching series and movies on TV and greater increase in using social networks were associated with lower PHI score. A greater increase in the consumption of series and movies was specifically related to lower scores in hedonic and experienced wellbeing. Moreover, a higher increase in the use of social networks was associated with lower eudaimonic, hedonic, and experienced well-being. The changes in other consumption habits did not show significant associations with happiness indicators.

**Table 4:** Pearson bivariate correlations between change in habits and happiness dimensions.

	General well-being	Eudaimonic well-being	Hedonic well-being	Social well-being	Experienced well-being	PHI
Change in watching TV: series and movies	−0.11 (−0.24, 0.02)	−0.12 (−0.24, 0.01)	−0.29^***^ (−0.40, −0.16)	−0.10 (−0.25, 0.04)	−0.14^*^ (−0.27, −0.01)	−0.19^**^ (−0.31, −0.06)
Change in watching TV: general	−0.07 (−0.20, 0.06)	−0.06 (−0.18, 0.07)	−0.13 (−0.26, 0.01)	−0.04 (−0.19, 0.11)	−0.07 (−0.20, 0.07)	−0.09 (−0.22, 0.05)
Change in listening to the radio	0.04 (−0.08, 0.15)	0.01 (−0.11, 0.10)	0.06 (−0.07, 0.19)	0.06 (−0.08, 0.19)	0.07 (−0.10, 0.23)	0.04 (−0.08, 0.15)
Change in following news	−0.01 (−0.14, 0.11)	0.03 (−0.08, 0.14)	−0.02 (−0.14, 0.10)	0.03 (−0.12, 0.17)	−0.04 (−0.16, 0.09)	0.01 (−0.11, 0.13)
Change in using SNS	−0.13 (−0.28, 0.03)	−0.14^*^ (−0.33, −0.08)	−0.20^**^ (−0.32, −0.06)	0.01 (−0.11, 0.12)	−0.20^**^ (−0.33, −0.05)	−0.17^*^ (−0.34, −0.01)

* *p* < 0.05;

** *p* < 0.01;

*** *p* < 0.001.

## Discussion

As findings of this exploratory study indicated, for all cases except for the radio, the daily media consumption during confinement at home because of the COVID-19 increased considerably, at least among the sample of participants.

With regard to the initial hypotheses, which linked this rise of media consumption to happiness and well-being, some different results were observed. In the case of H_1_, the consumption of fiction on TV and its positive association with happiness (PHI), was not supported by our data. Indeed, the resulting relationship was inverse. That is, the greater the consumption of fiction on TV during confinement and the greater the increase in that consumption with respect to pre-confinement, the subject reported less happiness (PHI). The same direct relationship was found between general entertainment on TV during confinement and happiness, but not between happiness and the increase in that behavior compared to pre-confinement; thus, our results partially supported H_2_.

TV use is mostly a passive habit, from a physical perspective, it encourages a sedentary lifestyle that is detrimental to both physical and mental health (Shiue, 2015). At a Cognitive level, fiction and general entertainment on TV do not involve any intellectual effort, which could not fulfill expectations in terms of well-being. The reward that this use provides may reward at a very short term or even decrease well-being.

Thus, Gui and Stanca (2009) consider TV consumption as a clear example of an overestimation of the reward obtained for a self-determined behavior, which could be related to its possible addictive component. In this sense, an easy and immediate relaxation is obtained, with little or no involvement by the consumer.

A possible cause of the decrease in well-being as a consequence of time spent watching TV could be the excessive availability of channels, contents, and possibilities that the “small” screen offers nowadays. Here, the evidence of the relationship of the overabundance of consumption choices in many product categories and happiness has been already pointed out (Schwartz, 2005). The same consequence was observed in relation to the oversupply of channels, content, and OTTs, as concluded by Gui and Stanca (2009), or Benesch et al. (2010), particularly among intense TV viewers.

Although the negative relationship between TV consumption and happiness seems clear, its causality is not, as pointed out by Bayraktaroglu et al. (2019). However, thanks to the possibilities of assessment of experienced well-being provided by PHI, when exploring the activities performed the previous day, added to the conditions of confinement that implied a certain “freezing” of life, it seems reasonable to estimate some causality between the activities undertaken the previous day and the hedonic and eudaimonic state at the time of the survey.

When we face psychologically adverse situations, as Taquet et al. (2016) pointed out, it seems natural to seek refuge in pleasant short-term activities, which have an immediate effect, although this has its dangers. An example would be watching TV passively and excessively, without greater emotional or cognitive involvement. The benefits of this type of activity are so short term that it is plausible to expect the emergence of a negative causal spiral. We would, therefore, be far from what is known as an optimal experience (Csikszentmihalyi, 1990), which besides reducing the time available for other more profitable (in terms of hedonia and eudaimonia) activities, and they clearly present risks for both physical health, due to sedentarism, and mental health, i.e., depressive symptoms (Bin et al., 2019).

Furthermore, there was no significant relationship, either positive or negative, between daily consumption of news and happiness, regardless the media. No association was neither observed concerning the change between news consumption before and during confinement, so that H_3_ was not supported by our data. In other words, neither more exposure to the news, nor a greater increase in relation to pre-confinement, was associated with neither less nor more happiness. This is contrary from what was found in other studies already mentioned (Johnston and Davey, 1997; Havrylets et al., 2013; Masip et al., 2020), but is consistent with works such as those by Cuñado and Pérez de Gracia (2012), who found a negative effect of TV on happiness, but they did not find that association with reading news and newspapers. Robinson and Martin (2008) suggested that the happiest people were those who spent less time watching TV and, conversely, more time reading newspapers. Also, Hall (2016) found that among those who spent more time on the Internet looking for information, those seeking news content scored higher in happiness, also with eudaimonia.

Perhaps an explanation for the fact that a high news consumption during the confinement did not imply a lower happiness could be the proliferation and bombardment of bad news (health, social, and economic issues), which may serve to compare the global situation with the individual in positive terms. As mentioned above, most of the sample was not experiencing the worst consequences of the tragedy directly, what fortunately could be extrapolated to the general population. Thus, the comparison would be positive. In fact, the questionnaire also explicitly asked if the respondent considered his or her own circumstances better or worse than the global situation lived in the worldwide. The results indicated that the majority, almost 70%, considered their circumstances better than the others’ circumstances.

Regarding the time spent listening to the radio, within the sample, there was no significant increase in the number of consumption minutes during confinement at home. Apart from this, the results showed that a higher level of radio use does not implied a higher level of happiness (H_4_), neither lower. It should be noted that this is the media whose reception is the least passive, compared to, for example, TV. That is, in the vast majority of cases, the radio listener is doing something else while listening. If before confinement, for example, radio was listened while commuting to work, during confinement, the people could listen to the radio, for example, while exercising, cooking, or doing other housework (Rodero, 2020), which somehow allowed him or her to feel in the company of others. As Rodero (2020) states, the radio is considered the closest media, which simulates companionship and drives away the feeling of loneliness, something that in many circumstances of confinement may have been important. In any case, the effect of listening to the radio on happiness level could be mediated by those other tasks or activities carried out while people listen to the radio.

With respect to the last hypothesis (H_5_), results clearly supported it. That means, the greater the use of SNS, the lower the happiness rate. In addition, the greater the increase in use during confinement with respect to consumption prior to it, the lower the level of happiness. This result is also in line with other studies prior to confinement (Arampatzi et al., 2018). Among the explanations for this result, there are several possibilities. The problem of social comparison has already been mentioned. Networks facilitate social comparison, which under normal conditions tends to have adverse effects on the happiness of individuals who see their lives as less exciting than what is apparent from the profiles of many of their contacts (Ayala et al., 2017). Bollen and Gonçalves (2018) assert that, in spite of social media apparently satisfying an essential human need, in terms of social relations, their use can lead to higher levels of psychological and social dysfunction. The aforementioned social comparison could be one of the main reasons.

But in addition to all this, and other disorders caused by SNS abuse, also mentioned above, the decline in happiness associated with it during confinement may have been driven by additional factors. For example, the frustration or nostalgia of seeing situations, places and events that were left behind and forbidden *sine die* due to confinement. Secondly, the impotence of seeing contacts in the timeline who are also known in real life, and with whom one could not be or meet face-to-face. Thirdly, the unease generated by growing social and political polarization and tension as a result of the pandemic and its management by the public authorities. A tension that, at least in Spain, has been considerable. In this sense, Hong and Zhang (2020) found that the influence of exposure to news, e.g., political news, on happiness was not the same whether the exposure was through traditional or digital means (e.g., social networks or electronic devices). Thus, traditional media increased the level of happiness, while digital media decreased it. Both effects, however, were mediated by other variables. In the case of traditional media, their positive effect on happiness occurred through the enhancement of public trust in government (GT), while the negative effect of new media on happiness occurred through the increase of perceived social risks (PSR). These results are intriguing, given the nature of contemporary Chinese society and its pattern of government intervention in the media. However, this dichotomy could be extrapolated to the data presented here.

The time spent in SNS, similar to that devoted to the TV, has led to less time available for other types of activities or habits that may positively correlate with well-being during confinement (i.e., more active lifestyles, both physically and cognitively, are more rewarding). This is the case, for example, of sport and physical exercise (Schuch et al., 2018), or reading (Billington, 2011).

As Twenge (2019) points out, perhaps the issue is not the use of SNS, but the excessive use of them. As Mochón (2018) suggests, excessive consumption could imply less well-being as an indirect consequence of displacing other activities that are more beneficial, related, for example, to sleep time, face-to-face social interaction, and upward social comparison (Twenge, 2019). In short, it seems clear that a greater well-being level does not seem to correlate with the increase in SNS consumption, as well as a higher TV consumption. In other words, a more moderate consumption of both media could imply a higher level of happiness, both in terms of hedonic, eudaimonic, and social well-being.

The present study is not exempt from some limitations. Among them, it should be acknowledged that variables such as personality traits may have a relevant impact (Lu and Hu, 2005). With regard to the sample, it would have been desirable to reach a greater number of respondents. In addition, in order to be able to generalize the results to the Spanish population, a study with a representative sample using probability sampling is necessary. In this sense, snowball sampling is a convenience sampling method usually used when it is difficult to access the sample under study, as is the case in this study (Naderifar et al., 2017). It could be interesting to combine the measurement of happiness with some other instrument that complemented the 22 items of PHI, but the fact is that this would have risked, making the questionnaire excessively long, possibly reducing the number of participants. PHI is a more complete and richer measure than many others, as discussed above, which covers different aspects of human happiness. Like all self-reports, there is also a risk of social desirability bias. However, the fact that the questionnaire was completely anonymous and remote may partly reduce this risk.

A future line of research, beyond the situation of confinement, should be to further explore causality in the relationship between media consumption and happiness. Given that the design of the study is cross-sectional, we can only draw conclusions based on the associations between the variables, without knowing the directionality of the effects, which requires a longitudinal design, nor the causality, which requires experimental manipulation. As pointed out by Bayraktaroglu et al. (2019), the possible causality between the two is still a matter of discussion. The heterogeneity of means, uses, situations, etc., raises the need to combine studies with other types of methodologies.

Finally, it is worth mentioning that among the comments that many respondents left after completing the questionnaire, almost all were positive. They said that they had enjoyed the questions and that many of them had raised interesting issues that were not previously noticed. This served, at least, to ensure that the development of this research itself did not imply any harm, but that even the subjects could have some benefit, at least for a few minutes, from their long confinement.

## Conclusion

The main conclusion of this study suggests that the higher media consumption did not seem to help so mucho to well-being and happiness, specially TV and Social Networks. However, it is important to avoid demonizing either of these media. The effects of SNS, TV, and the media as a whole on well-being, like almost every human tool, depend on quality and quantity of use. A rational and rationed use, as Mochón (2018) points out, may not diminish happiness but rather have positive effects. These can even be used as means to enjoy not only hedonic happiness, but also to exercise eudaimonia, as shown by various interventions from Positive Psychology (Niemiec and Wedding, 2014; Rieger et al., 2014; Yu, 2020).

Thus, the proposal could be a more moderate in time terms, but also a more virtuous media consumption. That is, going beyond mere hedonic entertainment and looking for more eudaimonic enjoyment, more rigorous information media, etc. We should remember once again that hedonia and eudaimonia need each other (Huta, 2015), that happiness and virtue nourish each other in a virtuous cycle (Kesebir and Diener, 2013), as Aristotle or Seneca (2018) argued hundreds of years ago.

If all this is true in normal times, it may be also true in times of confinement and pandemic, as also Eden et al. (2020) point in their recent study. In the late Middle Ages, Bocaccio (2013) wrote his famous Decameron. He narrated how 10 young people fled from the plague that devastated Florence and took refuge, confined, in a beautiful country villa. To pass the days, they combined routine tasks with storytelling sessions. They did this for entertainment, but also to draw lessons from each story. Just like them, in our confinement days, we had to learn to entertain ourselves, and to do so in a way that would also make us genuinely happier. Nowadays, a personal media environment should really serve as an ally for such aim.

## Data Availability Statement

The raw data supporting the conclusions of this article will be made available by the authors, without undue reservation.

## Ethics Statement

The studies involving human participants were reviewed and approved by Ethics Committee from Universidad Loyola Andalucía. The patients/participants provided their written informed consent to participate in this study.

## Author Contributions

JM-V was involved in the conceptualization, design, data collection, and writing of the manuscript. DG-B was involved in the conceptualization, planning, and data analysis. JL was involved in writing and reviewing of the manuscript. All authors contributed to the article and approved the submitted version.

### Conflict of Interest

The authors declare that the research was conducted in the absence of any commercial or financial relationships that could be construed as a potential conflict of interest.

## Footnotes

^1^www.qualtrics.com
